# HSP60 mimetic peptides from *Mycobacterium leprae* as new antigens for immunodiagnosis of Leprosy

**DOI:** 10.1186/s13568-023-01625-9

**Published:** 2023-10-27

**Authors:** Mayara Ingrid Sousa Lima, Meydson Benjamim Carvalho Corrêa, Emilly Caroline dos Santos Moraes, Jaqueline das Dores Dias Oliveira, Paula de Souza Santos, Aline Gomes de Souza, Isabela Maria Bernardes Goulart, Luiz Ricardo Goulart

**Affiliations:** 1 Laboratory of Genetics and Molecular Biology, Department of Biology, Federal University of Maranhão, São Luís, MA Brazil; 2 Postgraduate Program on Health and Environment and Postgraduate Program on Health Sciences, Federal University of Maranhão, São Luís, MA Brazil; 3 Laboratory of Biotechnology, Federal University of Tocantins, Palmas, TO Brazil; 4 Institute of Biotechnology, Federal University of Uberlandia, Uberlandia, MG Brazil; 5 National Reference Center in Sanitary Dermatology and Leprosy, School of Medicine, Clinics’ Hospital, Federal University of Uberlandia, Uberlandia, MG Brazil; 6 Department of Medical Microbiology and Immunology, University of California, Davis, CA USA

**Keywords:** Phage display, IgG antibody, Chaperonin, Lepromatous patients

## Abstract

The early diagnosis of leprosy serves as an important tool to reduce the incidence of this disease in the world. Phage display (PD) technology can be used for mapping new antigens to the development of immunodiagnostic platforms. Our objective was to identify peptides that mimic *Mycobacterium leprae* proteins as serological markers using phage display technology. The phages were obtained in the biopanning using negative and positive serum from household contacts and leprosy patients, respectively. Then, the peptides were synthesized and validated *in silico* and in vitro for detection of IgG from patients and contacts. To characterize the native protein of *M. leprae*, scFv antibodies were selected against the synthetic peptides by PD. The scFv binding protein was obtained by immunocapture and confirmed using mass spectrometry. We selected two phase-fused peptides, MPML12 and MPML14, which mimic the HSP60 protein from *M. leprae*. The peptides MPML12 and MPML14 obtained 100% and 92.85% positivity in lepromatous patients. MPML12 and MPM14 detect IgG, especially in the multibacillary forms. The MPML12 and MPML14 peptides had positivity of 11.1% and 16.6% in household contacts, respectively. There was no cross-reaction in patient’s samples with visceral leishmaniasis, tuberculosis and other mycobacteriosis for both peptides. Given these results and the easy obtainment of mimetic antigens, our peptides are promising markers for application in the diagnosis of leprosy, especially in endemic and hyperendemic regions.

## Key points

PD technology could be used to get and characterize *M. leprae* mimetic peptides;

MPML12 and MPM14 peptides detect IgG antibody in leprosy patients;

HSP60 mimetic peptides are specific for leprosy diagnosis.

## Introduction

Despite the high reduction in cases in recent years, leprosy remains a serious public health problem, especially in developing countries such as India, Brazil and Indonesia. The treatment, early diagnosis and monitoring of household contacts are essential elements for leprosy control and prevention of disease progression (WHO 2021a). Additionally, improving the diagnosis of leprosy can aid in the correct clinical classification and treatment, preventing recurrences and controlling infection (Araújo et al. 2012).

The complementary immunological tests available for leprosy, which include the use of natural *Mycobacterium leprae* antigens, have represented an important tool in the diagnosis of the disease, as PGL-1 (Leturiondo et al. 2019), recombinant proteins (Reece et al. 2006; Silvestre et al. 2018), or fused proteins (Silva et al. 2017; Silvestre et al. 2020) have been used in ELISA (enzyme-linked immunosorbent assay) and lateral flow tests (Bührer-Sékula et al. 2007).

Antigens from the Heat Shock Protein (HSP) family, produced under stress conditions and analogous to eukaryotic GroEL and GroES proteins, such as HSP65 or ML0317, have also shown potential application in tests for the diagnosis of leprosy (Laminet et al. 1990; van Eden et al. 2013). It is known that such proteins are abundant in the membrane of the bacillus, influence in immunological recognition mediated by T lymphocyte precursors, and are involved in processes of altered recognition and presentation of antigens (Wiker et al. 2011). Although obtaining native antigens is difficult, it is possible to use mimetic peptides as biomarkers because they are small molecules, easy to obtain and synthesize, capable of mimicking natural pathogen antigens while maintaining their specificity and reactivity (Goulart et al. 2010).

The *phage display* (PD) technology has become a widely used methodology for the selection of peptides and antibodies, representing a promising alternative for identifying immunogenic proteins (Kügler et al. 2013), in addition to being a technique capable of selecting peptides with specific binding domains (Sundell and Ivarsson 2014). Thus, these peptides obtained by PD can detect antibodies forming an antigen-antibody (Wang et al. 2019).

Recently, our group demonstrated that the synthetic peptide PGL1-M3, obtained by PD, which mimics epitopes of the PGL-1 of *M. leprae*, has promising application in different immunoassay platforms, and could become a substitute for the native antigen (Yotsumoto Neto et al. 2019; Lima et al. 2020). Therefore, this work demonstrates the use of *M. leprae* HSP60 mimetic peptides, which detect IgG in patients and contacts, as a tool for diagnosis of leprosy, which can be used for different immunological platforms.

## Materials and methods

### Biological samples

The volunteers were recruited in the State of Minas Gerais, Brazil. Patients were classified according to Ridley and Jopling (Ridley and Jopling 1966) and blood samples collected during the diagnosis. Household contacts’ samples were collected during monitoring of patients. Serum samples of newborns (n = 10) without maternal history of leprosy were used as true negative controls. For specificity tests, visceral leishmaniasis and pulmonary tuberculosis patients’ sera were used.

### Phage display (peptides library)

**Fig. 1:**
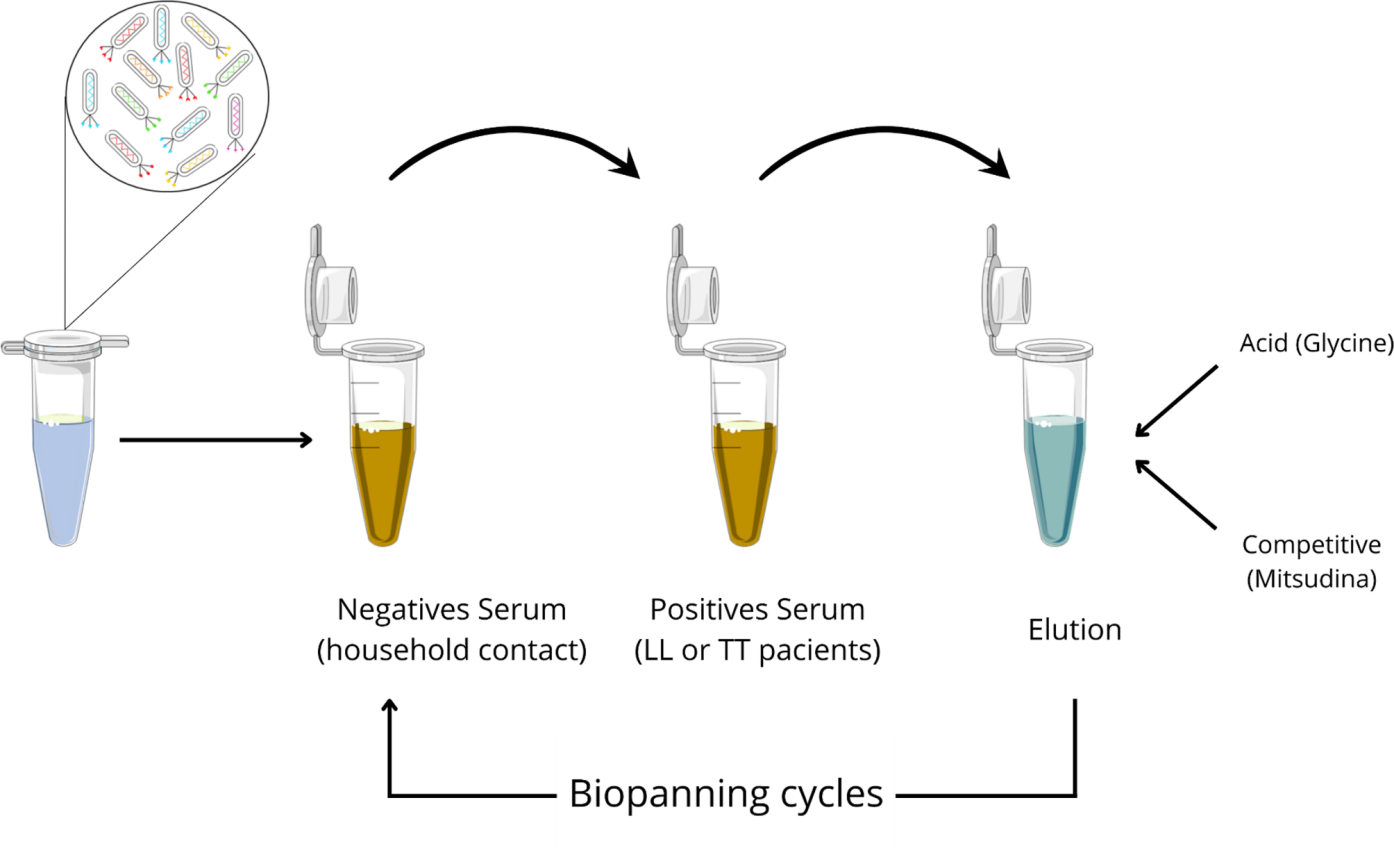
Biopanning scheme. TT (tuberculoid patients); LL (lepromatous patients)

The Ph.D.-C7TM library (New England BioLabs® Inc.) was used to perform the biopanning, targeting the purified IgG antibody from tuberculoids (TT), lepromatous (LL) and household contacts (HC). Purification was performed using anti-human IgG-specific γ-chain resin (Sigma-Aldrich). The liquid biopanning was performed in resin used for IgG purification, as described by (Barbas et al. 2001) and shown in Fig. 1. In each cycle, totally 05 cycles, phages were amplified and titrated in an *Escherichia coli* ER2738 culture. The selected phages, obtained from the non-amplified cycle, were used for DNA extraction and sequencing.

### Phage ELISA

To validate the peptides expressed on the surface of phages, an ELISA was performed with pools of serological samples from groups TT, LL and HC. Microtiter plates (Polysorp™) were sensitized with anti-M13 antibody (1 µg) and blocked with PBS-BSA 5%. A 50µL/well of phage supernatant was added, followed by the pool of IgG (1:100). Anti-human IgG (1:5000) with peroxidase was used as a secondary antibody for the detection of the immune complex.

Phage V06 (GenBank accession number: OQ835552) and V13 ((GenBank accession number: OQ835553) were tested with 40 samples of TT, LL and HC, following the validation protocol, with the exception of the addition of purified phage (1 × 10^10^) and serum, using individual serological samples (1:100).

### Bioinformatics and peptide design

The *in-silico* deduction of the amino acid sequences was conducted through the online Expasy Translate Tool (Gasteiger et al. 2003). Modeling of the synthetic peptide, obtained by commercial synthesis (Peptide 2.0) and GroEL protein was done using software I-TASSER, and the molecular structures were visualized using PyMOL 2.5. The alignment of the peptides and the three-dimensional structures of the proteins were performed using the Pepsurf software (Mayrose et al. 2007).

### Synthetic peptides ELISA

High affinity plates (Maxsorp - Nunc®) were sensitized with the synthetic peptides (1 µg), MPML12 or MPML14. Plates were incubated overnight and blocked with PBS-BSA 5%. For detection of IgG, serum (1:100) from 55 contacts and 135 patients was used. Anti-IgG secondary antibody with peroxidase (Sigma-Aldrich) was used following manufacturer’s instructions.

### Phage Display (antibody library)

We performed three individual biopanning of antibodies binding to synthetic peptides using a scFv library fused to PIII protein (Barbas et al. 2001). The scFv combinatorial library built from a healthy individual was amplified with the aid of the helper phage, and titrated.

After five successive washes, bound phages were eluted with glycine (0.2 M; pH 2.2). The eluded phages were amplified in *Escherichia Coli* XL1-Blue for plasmid extraction using a Miniprep Kit (Qiagen-27106), followed by electroporation in *E. coli* TOP-10 F’. Transformed bacteria were plated with SB medium, 2% (v/v) of 2 M glucose and 2.5mM IPTG (Sigma-I6758), to obtain soluble scFv. The most reactive scFv antibody was purified in a Nickel affinity column (Histrap HP 5mL; GE Healthcare). Then, a plate was sensitized with the supernatant containing soluble scFv, blocked with PBS-BSA 5% and detected with the anti-hemagglutinin (anti-HA) coupled with peroxidase (1:2500). The most reactive scFv was used for ELISA tests against specific targets.

After obtaining the most reactive scFv against each peptide, the antibodies were purified on a Nickel affinity column (Histrap HP 5ml, GE Healthcare) on HPLC (ÄKTApurifier-GE Healthcare) and concentrated by lyophilization.

### Antibody validation in ELISA

To detect the expressed antibodies, plates were sensitized with the supernatant containing the soluble scFv and blocked with PBS-BSA 5%. Anti-HA conjugated to peroxidase (1:2500) was used as a secondary antibody.

After the ELISA assay, tests were performed to detect interaction with the target. The plate was sensitized with 1 µg of the peptides and blocked with PBS-BSA 5%. Supernatant containing the scFv was placed in contact with the plate, followed by the same steps used in the previous ELISA.

### Antibody sequencing and bioinformatics

Sequencing of the light (mmb4 *primer*) and heavy (mmb5 *primer*) antibody chains was performed using a DyEnamic ET Dye Terminator Cycle Sequencing kit on MegaBaceTM 1000 (GE Healthcare). The deduction of anti-MPML12-E3 (GenBank accession number: OQ835554) and anti-MPML14-G2 E3 (GenBank accession number: OQ835555) antibody sequences was performed in the IgBlast (Ye et al. 2013) and Vbase2v (Retter et al. 2005) software. The 3D structure was obtained using I-TASSER (Yang et al. 2014b) and Kotai Antibody Builder (Duhovny et al. 2002). Validation of the binding affinity of the anti-MPML14 antibody with synthetic peptide MPML14 and the GroEL protein was performed in PatchDock (Schneidman-Duhovny et al. 2005).

### Immunoprecipitation and mass spectrometry

To find out which *M. leprae* native protein the MPML14 peptide mimics, the scFv anti-MPML14 antibody was coupled to Ni-charged MagBeads (GenScript) magnetic nanoparticles according to the protocol described by the manufacturer. Subsequently, the total protein extract of *M. leprae* (1000 ug/ml) was placed in contact with the nanoparticles coupled to the antibody of interest for 1 h at room temperature. Washes (10x) were performed to remove non-binding proteins. Antibody-bound proteins were eluted with glycine acid (0.2 M pH 2.2). The protein present in the eluate was identified using mass spectrometry.

Spectrometry analyses were performed by reduction and alkylation with DTT and iodoacetamide, enzymatic digestion with trypsin and liquid chromatography in HPLC coupled to Quadrupole-Time of Flight electrospray (LC-ESI-Q-TOF). Equipment calibration was performed to 10 ppm precision and a resolution of 9300 for ion m/z 588,8692.

### Statistical analysis

Statistical analyses of the data were performed using the GraphPad prism software version 9 (GraphPad Software, San Diego, CA, USA). To assess the chances of developing the disease or leprosy reaction, we performed a contingency analysis using Fisher’s exact test with calculation of odds ratio and confidence interval.

## Results

### Reactivity of peptides fused to bacteriophages

**Fig. 2:**
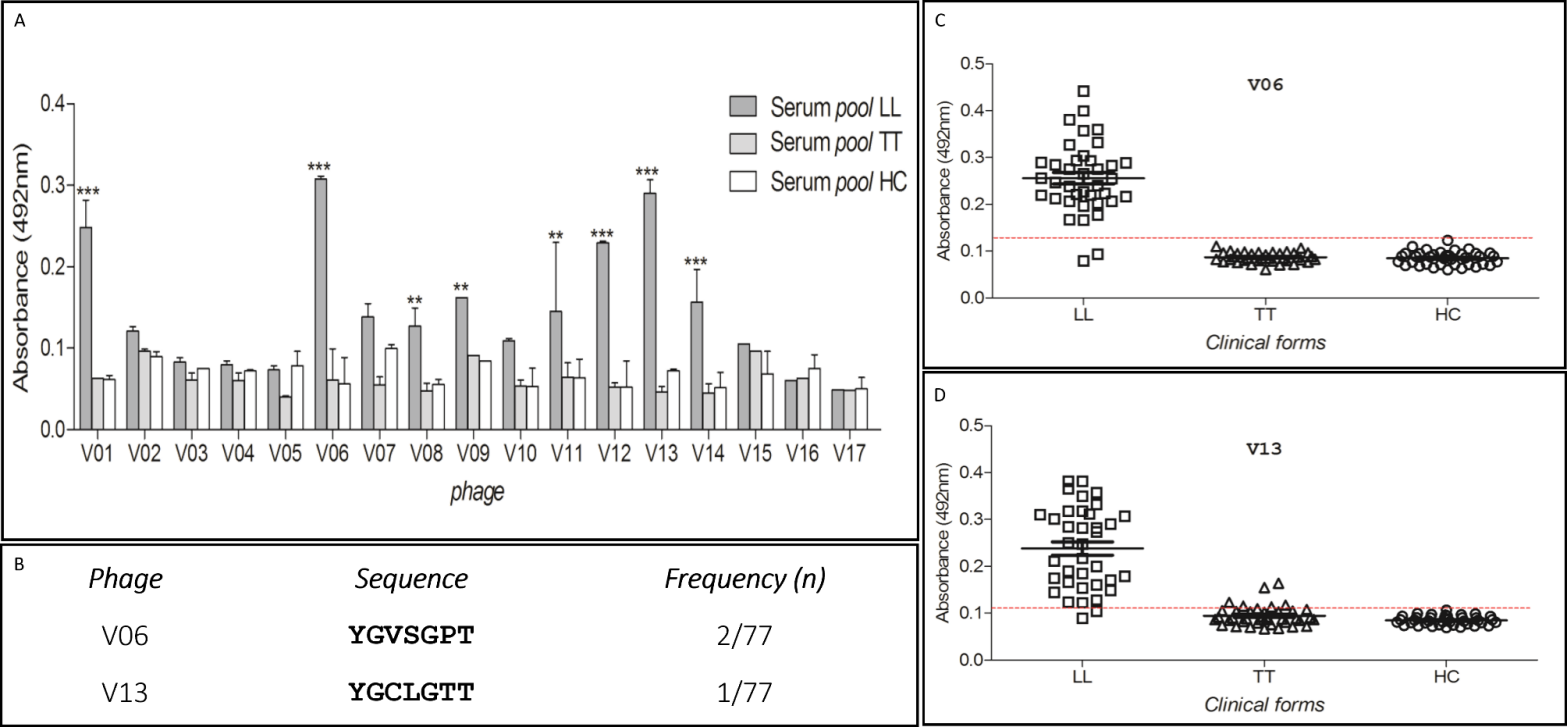
Phage reactivity. **A**, pre-validation of phage reactivity testing pools of serum from patients and contacts. **B**, sequence and frequency of peptides expressed on phages. **C** and **D**, reactivity of the two phages selected in the pre-validation testing individual serum. TT (tuberculoid patients); LL (lepromatous patients); HC (household contacts)

After biopanning, 77 clones were obtained (data not shown) and of these, 17 were used in the validation with serum pools. The phages V06 and V13 were the most reactive (Fig. 2A) when comparing patients lepromatous and contacts (p < 0.001) (Fig. 2 C-D). The amino acid sequences of peptides expressed on the surface of phages were presented in Fig. 2B.

### *In silico* validation of peptide sequences

**Fig. 3:**
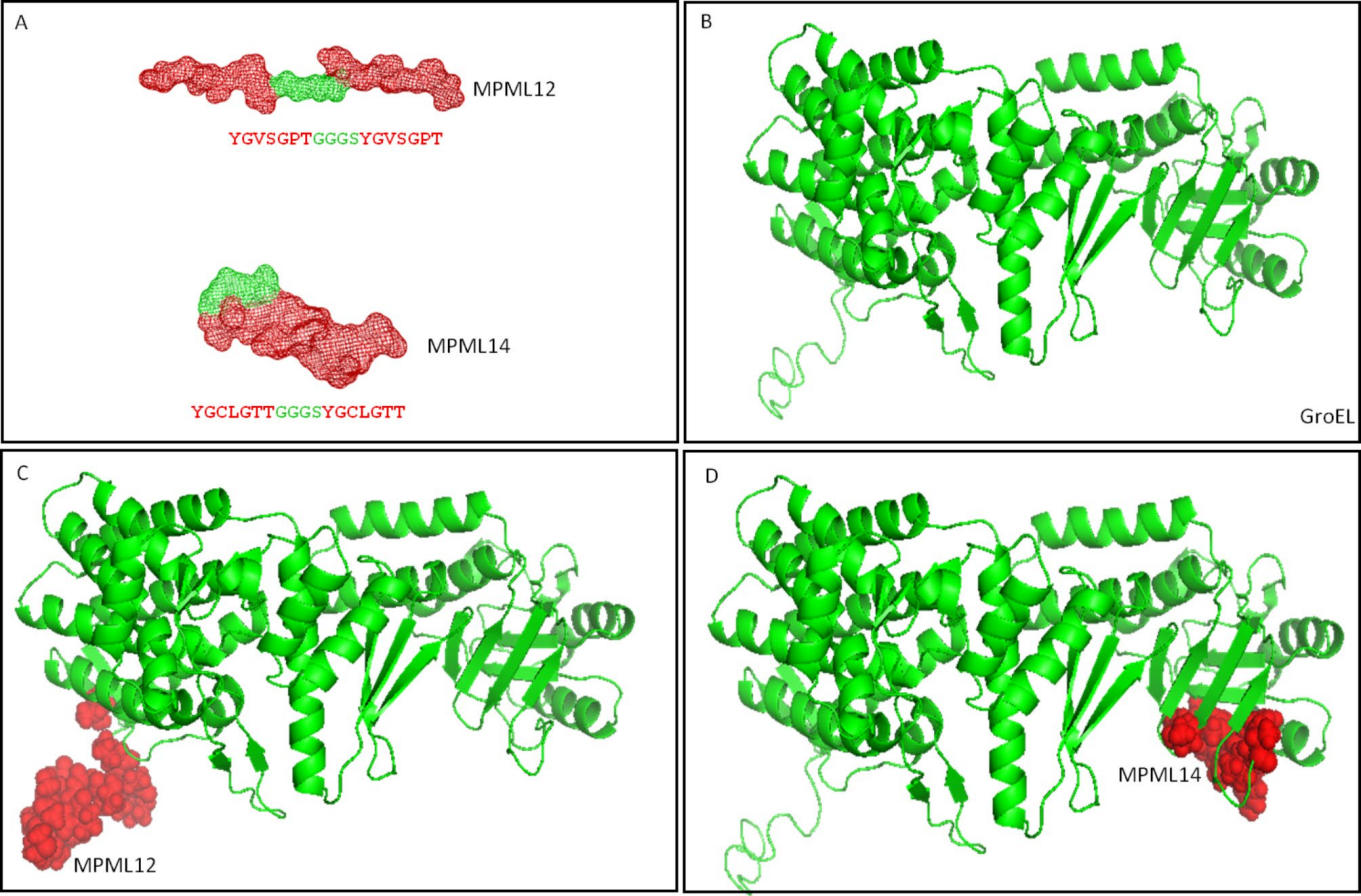
Design of synthetic peptides, GroEL from *M. leprae* and alignment. **A**, sequence and three-dimensional structure of synthetic peptides. **B**, GroEL from *M. leprae*. **C** and **D**, alignment of synthetic peptides with GroEL

From the prediction of amino acids present in peptides fused to phage proteins (V06 and V13), two synthetic peptides were designed - MPML12 and MPML14. The design of the peptides featured two repeats of the original sequences interspersed with the GGGS spacer. MPML12 maintained a linear three-dimensional structure, while MPML14 acquired its own conformational structure in globular shape (Fig. 3A). Peptides MPML12 (alignment on amino acid 488 to 494 of the protein), and MPML14 (alignment on amino acid 125 to 131 of the protein) aligned with the *M. leprae* GroEL protein in two different regions of the molecule (Fig. 3B-D), suggesting that they may be mimetics of this protein.

### Reactivity of peptides after chemical synthesis

**Fig. 4:**
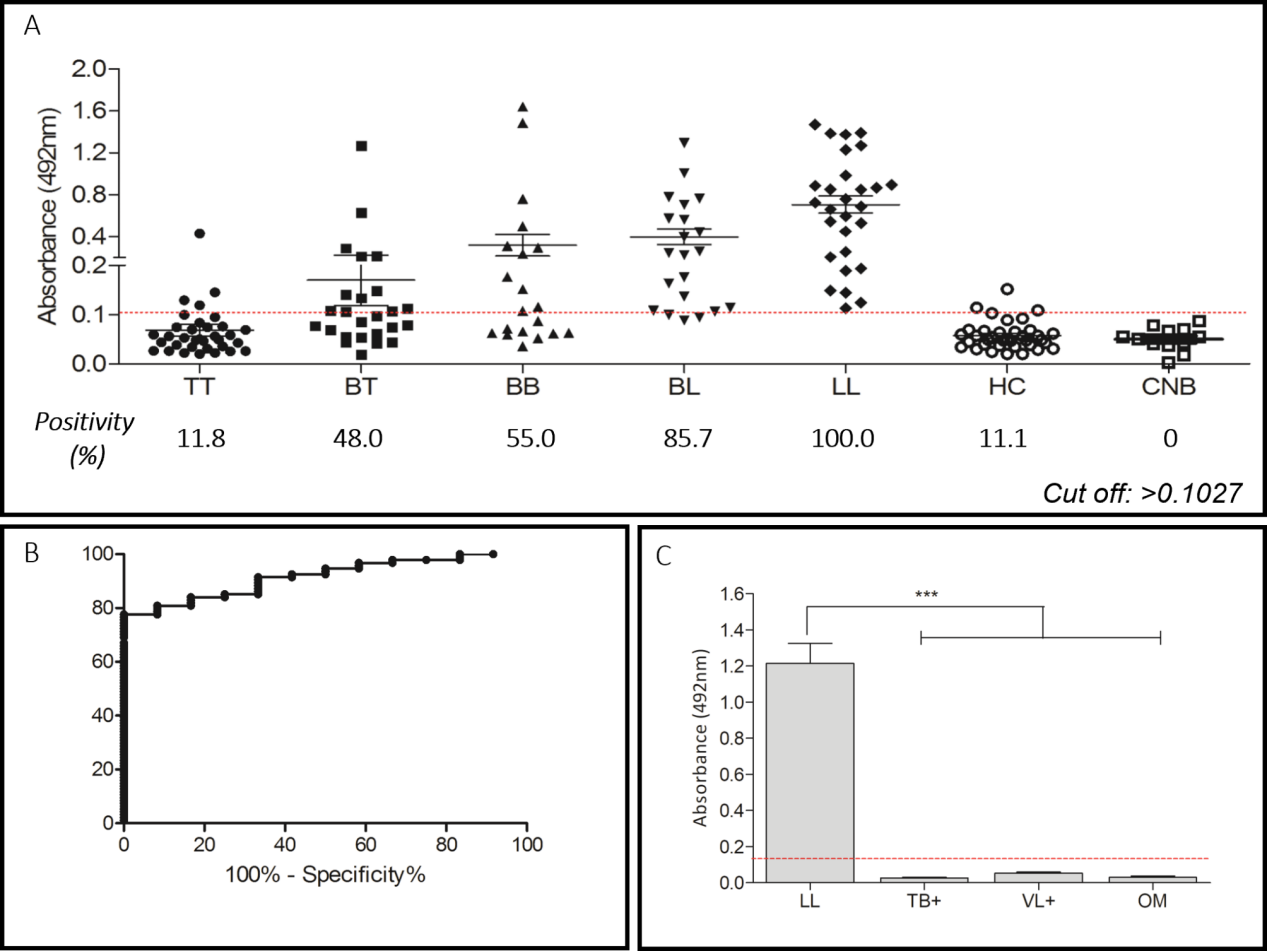
Detection of IgG antibodies using MPML12. **A**, MPML12 reactivity with patients of different clinical forms, household contacts and newborns, and positivity by clinical form. **B**, sensitivity and specificity. **C**, comparison of IgG detection in lepromatous patients and individuals with other diseases. TT (tuberculoid); BT (borderline-tuberculoid); BB (borderline-borderline); BL (borderline-lepromatous); LL (lepromatous); HC (household contacts); CNB (newborn control); Tuberculosis (TB+); Visceral Leishmaniasis (VL+); Other mycobacteriosis (OM)

The MPML12 peptide had the highest IgG titer in multibacillary patients, with positivity ranging from 11.8% for TT to 100% for LL, gradually increasing in the intermediate forms (BT: 48%, BB: 55%, and BL: 85.7%), with a sensitivity of 73.40% and a specificity of 100%. In contacts, positivity was 11.1% considering a cut off of 0.1027 (Fig. 4A-B). All controls, including newborns, patients with Visceral Leishmaniasis, Tuberculosis and other mycobacteriosis were negative for this antigen (Fig. 4C).

**Fig. 5:**
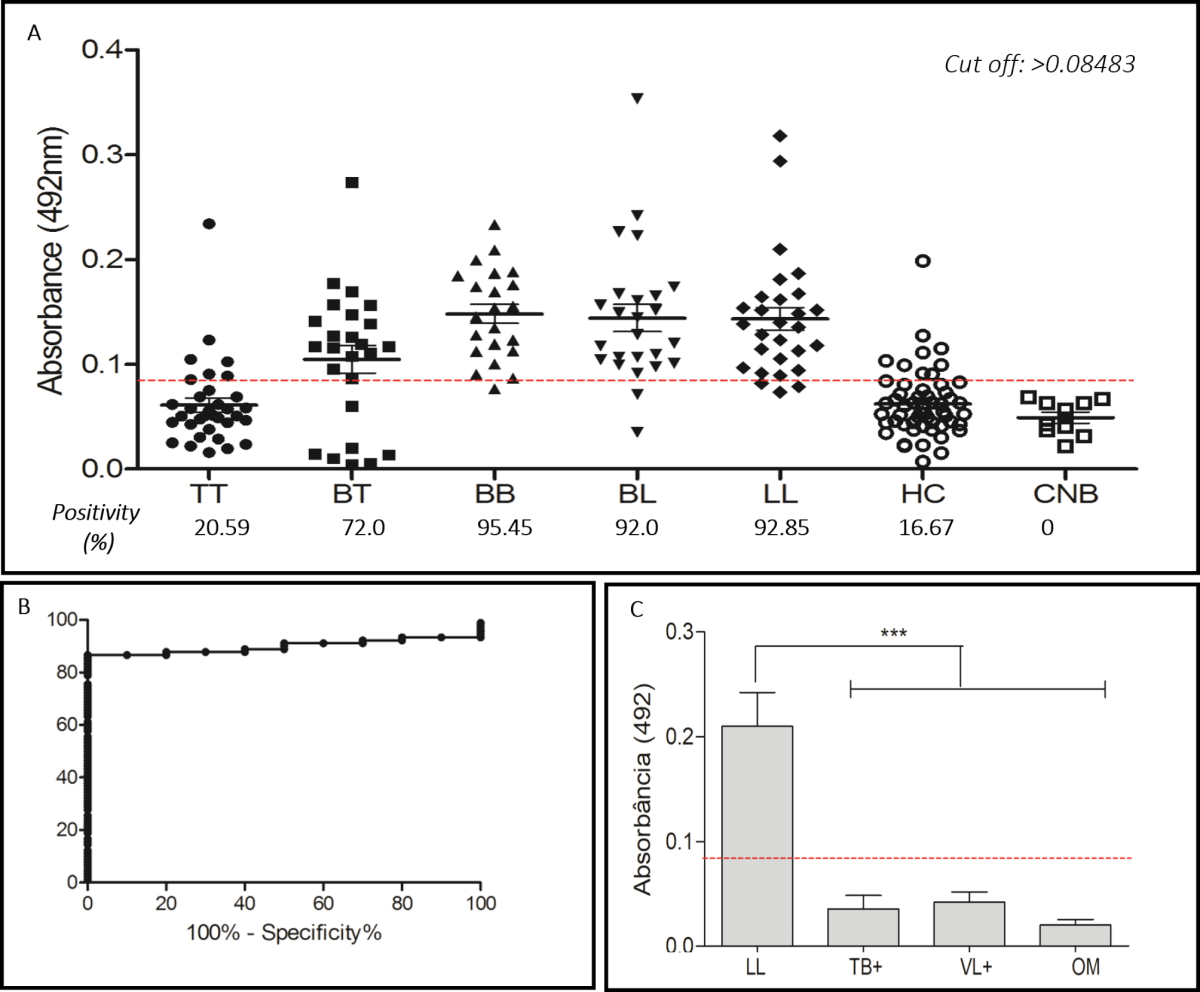
Detection of IgG antibodies using MPML14. **A**, MPML14 reactivity with patients of different clinical forms, household contacts and newborns and positivity by clinical form. **B**, sensitivity and specificity. **C**, comparison of IgG detection in lepromatous patients and individuals with other diseases. TT (tuberculoid); BT (borderline-tuberculoid); BB (borderline-borderline); BL (borderline-lepromatous); LL (lepromatous); HC (household contacts); CNB (Newborn Control); Tuberculosis (TB+); Visceral Leishmaniasis (VL+); Other mycobacteriosis (OM)

The MPML14 peptide showed a pattern similar to MPML12, demonstrating higher IgG titers in multibacillary patients. In LL, the positivity was 92.85%, lower than that found for MPML12. However, in the TT (20.59%) and BT (72.0%) groups, MPML14 detected a greater number of patients (Fig. 5A-B). Peptide reactivity in patients with visceral leishmaniasis, tuberculosis and other mycobacteriosis was below the cut off line and in newborn controls there was no positivity, with a specificity of 100% (Fig. 5C).

### Reverse engineering

**Fig. 6:**
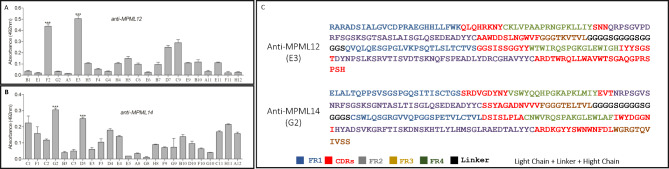
Anti-MPML12 and anti-MPML14 scFv antibodies. **A** and **B** show scFv antibodies expressed in *E. coli* top-10 *** p < 0.001. In C, sequence of expressed antibodies selected that recognize peptides MPML12 (E3) and MPML14 (G2)

To identify of which *M. leprae* antigen the peptides were mimetic, scFv antibodies were constructed against them. Figure 6 A and 6B show *E. coli* top-10 clones that expressed scFv specific for each peptide. More than one expressed antibody interacted significantly with the MPML12 and MPML14, the most reactive being selected: E3 (p < 0.001) and G2 (p < 0.001), respectively. Figure 6 C shows the light and heavy chain sequences of the two selected scFv.

**Fig. 7:**
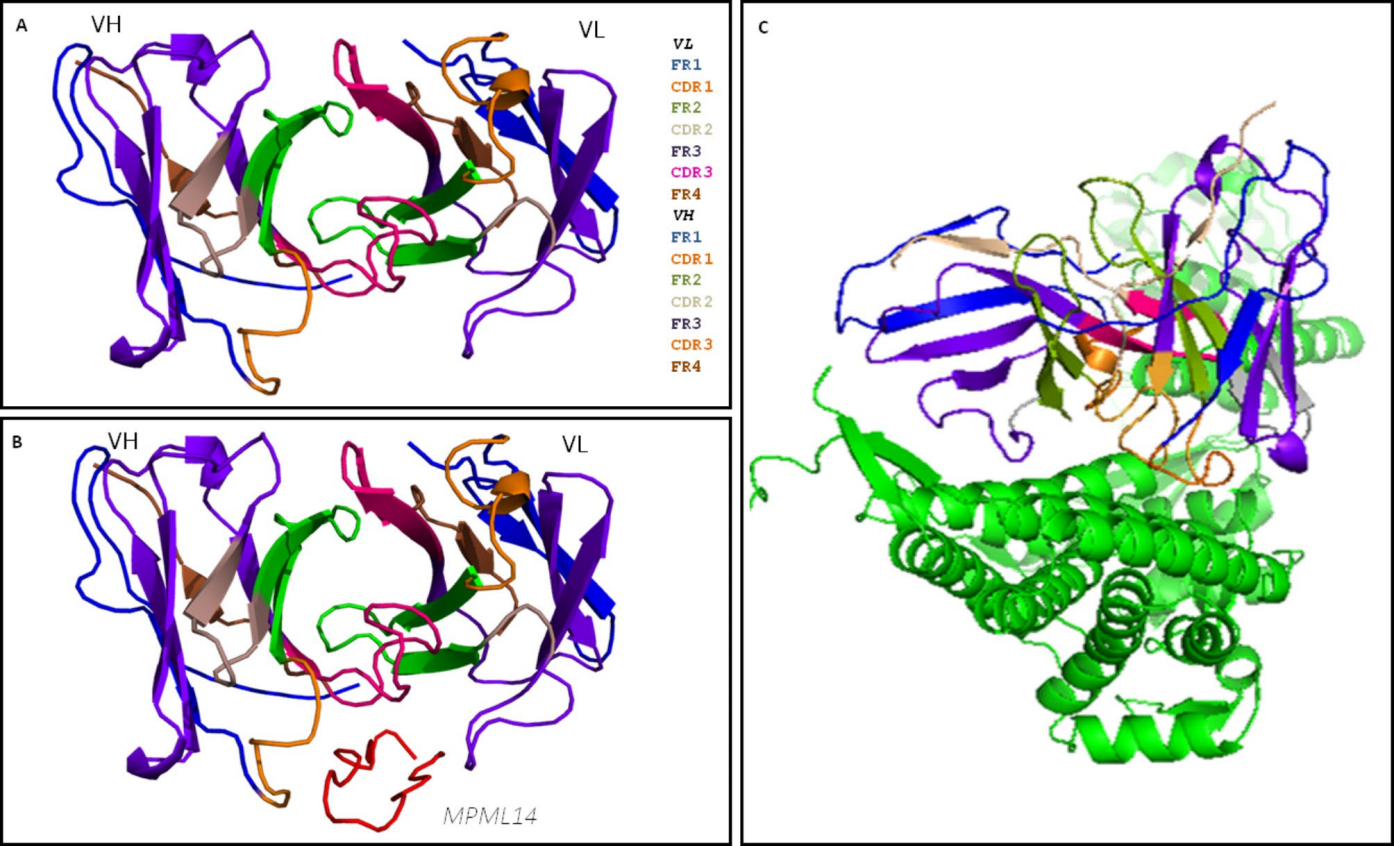
Bioinformatics of anti-MPML14. **A**, three-dimensional structure of anti-MPML14. **B**, interaction of anti-MPML14 with peptide MPML14; the heavy chain CDR3 region in pink, the heavy chain CDR1 region in orange, and the light chain FR3 region in purple. **C**, interaction of anti-MPML14 with GroEL; in orange, the heavy chain CDR1 region, and in blue, the light chain FR3 region, both close to amino acids 80 to 140 of the protein

The three-dimensional structure of anti-MPML14 is represented in Fig. 7A and analysis demonstrates that the interaction with peptide MPML14 occurs in the CDR1, CDR3 region of the heavy chain and in the FR3 region of the light chain, indicating that there is a binding affinity between peptide and antibody (Fig. 7B). In addition, anti-MPML14, through heavy chain CDR1 and light chain FR3, also demonstrated binding to the region located between amino acids 80 to 140 of the *M. leprae* GroEL protein (Fig. 7C).

Mass spectrometry results confirmed that the anti-MPML14 scFv antibody recognizes a 60 kDa chaperonin from *M. leprae*. This data proves, therefore, that the peptides obtained by phage display are mimetics of the 60 kDa heat shock protein and highlights that the antibody obtained specifically recognizes this protein from *M. leprae*.

## Discussion

In recent decades, there has been a decrease in the prevalence of leprosy in the world due to the use of multidrug therapy. Thus, the global strategy continues to be the reduction of new cases of the disease, especially those with grade II disability (WHO 2021b). The success of these strategies involves the development of new platforms that allow early diagnosis and prognosis, including new infection markers, contact monitoring and reaction predictors. In this sense, many studies aim to find and validate *M. leprae* antigens that can be used as markers with diagnostic applications (Reece et al. 2006; Geluk et al. 2009; Lobato et al. 2011; Hungria et al. 2012; Leturiondo et al. 2019).

Mimetic peptides are good candidates for biomarkers, especially as they are small molecules that mimic pathogen-specific antigens (Goulart et al. 2010). Phage display technology is a pioneer in selecting these peptides and has shown advantages in selecting specific molecules that are highly reactive against a variety of biological targets (Deroo and Muller 2001). The use of mimetic antigens for diagnosis by detection of circulating antibodies has been reported for pneumonia (Marston et al. 2002), tuberculosis (Yang et al. 2014a), neurocysticercosis (Manhani et al. 2011), leishmaniasis (Costa et al. 2014), anaplasmosis (Santos et al. 2012), hepatitis (Tan and Ho 2014) and leprosy (Alban et al. 2013; Lima et al. 2020).

The strategy of this work was to use IgG from leprosy patients as target, with the hypothesis that the selected peptides would hold great potential in recognizing these immunoglobulins. The importance of IgG for the diagnosis of leprosy has been described by several authors (Cabral et al. 2013; Alban et al. 2014). The success of the approach was evident with the reactivity of the peptides still expressed on the phage, where it was possible to select those (V06 and V13) that clearly differentiated LL and HC. It has been shown that the phage provides the peptide with an adequate conformational structure that helps in the antigen-antibody interaction during the selection process (Sundell and Ivarsson 2014).

During chemical synthesis, the conformation of the peptides must be preserved, as a structural flexibility can decrease the binding affinity of the peptides against their targets (Chen et al. 2014). To provide the conformation of the synthetic peptides, we used GGGS spacers, the same ones found to separate the peptide from the PIII protein in the phage (Barbas et al. 2001). Furthermore, duplication of the peptide’s amino acid sequence should amplify the antigen-antibody interaction site. From these strategies MPML12 and MPML14 preserved the reactivity that had been found in the phage.

Regarding characterization of the *M. leprae* natural antigen of which the peptides are mimetic, bioinformatics demonstrated the alignment with the heat shock proteins (HSP) GroEL and GroES. Microbial HSPs have been associated with the generation and induction of a Th1-type immune response (Rha et al. 2002). In contrast, GroES also induces high levels of IgG1 antibodies in leprosy patients across all spectrums of the disease (Hussain et al. 2004). Another study reported high levels of total IgG in response to GroEL and GroES in lepromatous patients, and low levels in the TT, BT and BB groups (Rojas and Segal-Eiras 1996). Just as these proteins would be related to the induction of IgG, our mimetic peptides were able to detect these antibodies in patients.

Mimetic peptides MPML12 and MPML14 demonstrated very similar results, with low reactivity in paucibacillary patients a gradual increase in the percentage of positives as they approach the lepromatous pole. This characteristic is very similar to that found for the PGL-1 antigen (Fabri et al. 2015; Do et al. 2018; Van Hooij et al. 2018; Leturiondo et al. 2019; do Carmo Gonçalves et al. 2020); and its synthetic derivatives ND-O-HSA (Lobato et al. 2011; van Hooij et al. 2018; Rumondor et al. 2019), NT-P-BSA and NT-P-HSA ML Flow (Bührer-Sékula et al. 2003, 2007; Moura et al. 2014; Ule Belotti et al. 2021). MPML12 and MPML14 detected, respectively, 100.0% and 92.85% of the LL group, while LID-1 (Duthie et al. 2011) and ML0405 (Duthie et al. 2007), detected 97.7% and 67.0%, respectively, in multibacillary patients. In addition, these two peptides can still be used as exposure markers, as they can detect positivity in contacts (MPML12- 11.1% and MPML14- 16.67%).

For diagnostic application of these mimetic peptides in leprosy, it is necessary to characterize the mimicked native antigens. For this, the production of scFv antibody fragments against peptides MPML12 and MPML14 was the strategy adopted to map the natural molecule. Antibody fragment libraries displayed on bacteriophages have been applied in epitope recognition (Chan et al. 2014). The anti-MPML12 and anti-MPML14 antibodies produced recognized their respective targets (peptides), corroborating the sequencing result, in which the produced antibodies had the important CDRs in antigen-antibody recognition. Synthetic libraries contain artificial CDR sequences built with the use of degenerate oligonucleotides which generate their great diversity (Shukra et al. 2014).

The *in-silico* analysis showed that anti-MPML14 binds to an immunogenic region of GroEL, corroborating the hypothesis that this peptide is a GroEL mimetic, as it is also aligned with this protein. However, we cannot discard the hypothesis that these peptides, MPML12 and MPML14, are mimetics of non-protein antigens, such as PGL-1, especially when analyzing the results of immunoreactivity, where the similar behavior of these molecules becomes evident.

The mimetic peptides from HSP60 obtained in this work are promising in the diagnosis of leprosy and should be used in the detection of IgG antibodies in patients and household contacts. Peptides MPML12 and MPML14 can be used especially for the diagnosis of multibacillary forms. The combined use of the peptides obtained in this work together with other *M. leprae* antigens can improve the complementary diagnosis of leprosy, especially in endemic regions in the world.

## Data Availability

The datasets generated during and/or analyzed during the current study are available from the corresponding author on reasonable request.
